# The spread model of food safety risk under the supply-demand disturbance

**DOI:** 10.1186/s40064-016-2881-2

**Published:** 2016-10-11

**Authors:** Jining Wang, Tingqiang Chen

**Affiliations:** School of Economics and Management, Nanjing Tech University, Nanjing, 211816 China

**Keywords:** Food safety risk, Supply-demand disturbance, Supervision behaviors, Spread model, Computational experiment

## Abstract

In this paper, based on the imbalance of the supply-demand relationship of food, we design a spreading model of food safety risk, which is about from food producers to consumers in the food supply chain. We use theoretical analysis and numerical simulation to describe the supply-demand relationship and government supervision behaviors’ influence on the risk spread of food safety and the behaviors of the food producers and the food retailers. We also analyze the influence of the awareness of consumer rights protection and the level of legal protection of consumer rights on the risk spread of food safety. This model contributes to the explicit investigation of the influence relationship among supply-demand factors, the regulation behavioral choice of government, the behavioral choice of food supply chain members and food safety risk spread. And this paper provides a new viewpoint for considering food safety risk spread in the food supply chain, which has a great reference for food safety management.

## Background

In a complete food supply chain from suppliers to consumers, any illegal economic behaviors occur in any one of the links from the raw materials suppliers to the food retailers, which will cause different kinds of food safety risk. In fact, food safety risk incidents are caused by many factors, including the food markets failures which is from the food markets’ externality, and information asymmetry (Resende-Filho and Hurley 2012; Kim and Kim 2013; Jones and Davidson 2014; Fellmann et al. 2014; Zissis et al. 2015; Levi and Zhang 2015; Zhang et al. 2015; Wang and Pallis 2014), and the mismatch between the costs and the benefits made the center of interest gravity shift toward the downstream of the industry, food companies paid too much attention to the market share, and the risks which were accumulated by the upstream interests contradiction to the extreme through the transmission of leverage effect (Lamboni and Azouma 2013; Hobbs et al. 2014), etc. In China, the accidents of food safety frequent occurred, which have led to consumers’ lack of social trust on food safety extremely (Wu et al. 2014). This reason is the lack of the perfect regulatory system and the perfect legal system in food safety supervision and consumer protection. Thus our paper will consider the selection of these relevant variables, including the sampling probability of the government in the production link and the retail link, the probability of taking legal action to defend their rights and interests after consumers find unqualified products, and the probability of winning after consumers take legal action and so on.

The others factors caused food safety risk and its spread including the interest game imbalance between all the main participant bodies in the food supply chain (Henry and Wernz 2015), the “adverse selection” of members of the food supply chain (Gil et al. 2015; Luning et al. 2015; LeBlanc et al. 2015) and “moral hazard” behavior of members of the food supply chain (Malekan and Dionne 2014; Wang and Pallis 2014; Weber 2015; Verrette 2015). These can lead to the spread effect of food safety risk in the food supply chain. Food safety risk existing in anyone of link in food supply chain. And which of any node of food supply chain would pass on the food supply chain to spread, and often present the amplification effects, then led to the occurrence of food safety incidents eventually (Bae et al. 2011; Hirschauer et al. 2012; Kirezieva et al. 2013; Boxstael et al. 2013; Manning and Soon 2013; Duret et al. 2014). From the perspective of food supply chain, which could realize that enhance the food supply chain’s ability to resist risks and effectively control food safety risks through establishing synergy mechanism between all the main participant bodies (Rong et al. 2011; Angeles Sanfiel-Fumero et al. 2012; Chen et al. 2014; Bruzzone et al. 2014; Eksoz et al. 2014; Migliore et al. 2015; Tang et al. 2015). However, the few aforementioned studies deeply investigate the propagation law of food safety risk in the food supply chain and its influential factors and mechanism. In fact, the unbalance of supply-demand and membership in the food supply chain can significantly affect food safety and its risk spread, and the members behavior. Therefore, in our paper, we will consider the effect of the characteristic of the supply-demand relationship and government supervision behaviors on the risk spread of food safety and the behaviors of the food producers and the food retailers to find out the propagation law of food safety risk in the food supply chain and its influential factors and mechanism. Our objective is to understand the influence of the supply-demand relationship and government supervision behaviors on the risk spread of food safety and the behaviors of the food producers and the food retailers.

The rest of this paper is organized as follows. “Second section” makes some assumptions and notations for the following investigation. “Third section” defines the contagious process and feature of food safety risk, and build an spread model of food safety risk in the supply chain. “Fourth section” uses numerical simulations to analyze the influence and active mechanism of the supply-demand relationship and government supervision behaviors on the risk spread of food safety and the behaviors of the food producers and the food retailers. Finally, the last Section summarizes some concluding remarks.

## Notations

The notation used in this paper can be summarized as follows:

- *D* is the food demand of consumers within a certain stage, and $D>0$;
- *R* is the food purchase orders of the food retailers to the food producers in the current, and $R>0$;
- *Q* is the maximum output of the food producers to provide qualified food, and $Q>0$;
- $P_{pr}$ is the trading price between the food producers and the food retailers; $P_{rc}$ is the trading price between the food retailers and consumers;
- $c_p$ is the purchasing cost of the raw material of the food producers, $c_{pp}$ is the food processing cost of the food producers, $c_{rs}$ is the food selling cost of the food retailers, $c_s$ is the food sampling cost of the food retailers. And $c_p>0$, $c_{pp}>0$, $c_{rs}>0$, $c_s>0$;
- $q_p$ is the raw material adulteration probability of the food producers, $q_s$ is the sampling probability of the food retailers. And $q_p>0$, $q_s>0$;
- $g_p$ is the sampling probability of the government in the production link of the food producers, $g_r$ is the sampling probability of the government in the retail link of the food retailers. And $g_p>0$, $g_r>0$;
- $\phi _p$ is the mean of the fraction defective of products that provided by the food producers within the nearly three phase, $\phi _r$ is the mean of the sampling rate of the food retailers within the nearly three phase. And $\phi _{p}>0$, $\phi _{r}>0$;
- $\alpha$ is the growth rate of food output when the food producers adulterate inferior materials. In other words, if one unit of food product is adulterated inferior materials, the food producers can obtain $1+\alpha$ unqualified products;
- $F_p$ is the unit penalty amount for the food producers after the government tests to the unqualified products in the production link of the food producers. $F_r$ is the unit penalty amount for the food retailers after the government tests to the unqualified products in the retail link of the food retailers;
- $\beta _1$ is the probability of taking legal action to defend their rights and interests after consumers find unqualified products, $\beta _2$ is the probability of winning after consumers take legal action;
- $\psi$ is the benefit of winning after consumers take legal action;
- $\theta$ is compensation ratio of the food producers to the food retailers if the unqualified products are found by the government in the retail link. In fact, for $\theta >0$, there will be collusion behaviors of the food producers and the food retailers. In this paper, We assume $0<\theta <1$. And $\theta$ is smaller, the smaller the degree of collusion between the food producers and the food retailers.

## The contagion model of food safety risk in the food supply chain

### The contagion mechanism of food safety risk

The food supply chain is mainly composed of the raw materials suppliers, the food producers, the food retailers, the consumers and the regulators. In the food supply chain, the illegal economic behaviors of the raw materials suppliers, the food producers, and the food retailers will cause kinds of food safety risk. However, the regulators regulate the economic behaviors of the raw materials suppliers, the food producers, and the food retailers. At the same time, the regulators also safeguard the rights and interests of all the behavior subjects. The regulators would ensure the orderly operation of the food supply chain. In the different stage, every supply chain members have their own behavior choice. However, some illegal economic behaviors will lead to food safety risk. In food supply chain, the behavior choice of each supply chain member as follows.

1. For the raw materials suppliers, they have two kinds of behavior choices. (1) Providing safe and qualified raw materials to the food producers. (2) Providing adulterated inferior materials to the food producers. In this case, the raw materials suppliers can provide more raw materials to the food producers, and would obtain more abnormal income. But this behavior will cause food safety risk, and transfer to the food producers.
2. For the food producers, their behaviors include two aspects. On the one hand, for behavior choice of the purchasing of raw materials. (1) Strict inspection for raw materials. But this behavior will increase the manufacturer’s purchasing cost. (2) Weak inspecting or no inspecting for raw materials. This would save the manufacturer’s purchasing cost, and increase the food supply. However, this behavior will cause food safety risk, and transfer to the downstream of the food supply chain. On the other hand, for behavior choice of the food production. (1) Strictly comply with the technology and standards of food production to process and make qualified product. (2) Illegal using additives, adulterating inferior materials, or simplifying the food processing craft to process and make product. This would greatly save the manufacturer’s production cost, and increase the food supply. However, this behavior will cause food safety risk, and transfer to the downstream of the food supply chain.
3. For the food retailers, they have two kinds of behavior choices. (1) Strict inspecting the upstream provided products of the food supply chain, and selling the qualified products to the consumers. (2) Weak inspecting or no inspecting the upstream provided products of the food supply chain. This behavior would greatly save the food retailers’ selling cost. But this will lead many products including the safety risk to be sell to the consumers.
4. For the consumers, they have two kinds of behavior choices in the face of unqualified or substandard food. (1) Depressed to go away and not taking legal action to defend their rights and interests after finding unqualified products. This behavior will indulge the upstream of the food supply chain to choice the risk behaviors. (2) Taking legal action to defend their rights and interests after finding unqualified products.
5. For the food regulators, their behaviors include two aspects. On the one hand, the behavior choice for supervising and managing of the food safety risk. (1) Strict inspecting every segment of the food supply chain, and severe punishment on illegal node in the food supply chain. This behavior will greatly increase the cost of illegality of the node, and restrain the occurrence of food safety risk. (2) Weak inspecting every segment of the food supply chain, and light punishment on illegal node in the food supply chain. This would lose the effect of supervise, and increase the incidence probability of food safety risk. On the other hand, the behavior choice for the prosecuting of the problem of food unsafe. (1) Positive dealing with complaint, and severely punish the illegal node in the food supply chain. This can effectively protect the interests of the related subject, and restrain the occurrence of food safety risk. (2) Negative dealing with complaint. This will lead to the food safety risk unrestrained spread in the food supply chain.

**Fig. 1:**
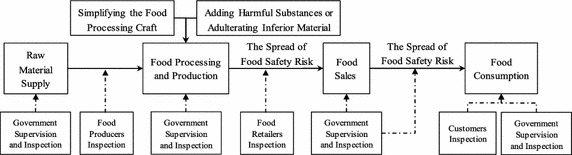
The spread sequence structure model of food safety risk in the supply chain

In this paper, we assume the food safety risk is caused by illegal using additives, adulterating inferior materials, or simplifying the food processing craft. In other word, the spread of the food safety risk from the food producers to the food retailers and the consumers in the food supply chain. Thus the spread sequence structure model of food safety risk in the supply chain as shown in Fig. 1.

### The model assumptions

According to Fig. 1, the supervision of food safety risk is a complex system engineering. In the process of the regulation, many market participants will be involved. In order to effectively analyze the contagion mechanism of the food safety risk in the food supply chain, we do some assumptions of the model as follows:

1. We assume the raw material suppliers are able to provide enough the needed raw materials of food producers. And there is no adulteration behavior of hazardous substances of the raw materials suppliers, the food producers and the food retailers in the food supply chain. In other words, the food safety risk is caused by illegal using additives, adulterating inferior materials, or simplifying the food processing craft in the process of food production and sales, rather than raw materials provider’s problem;
2. We assume the behaviors of illegal using additives, adulterating inferior materials, or simplifying the food processing craft can increase the level of supply, and the cost can be ignored;
3. We assume the food is easy to corrosion, and has high request to the shelf life. If there is no successful trading within the shelf life, the food must be scrapped;
4. We assume the food producers purchase raw materials and production quantity is based on the retailer’s orders;
5. We assume the contagion of food safety risk is no cross tiers and reverse in the food supply chain. In other words, the contagion of food safety risk is from the raw materials suppliers to the food producers, the food producers to the food retailers, the food retailer to consumers;
6. We assume that if the government found problems in the sampling inspection, the government punish only the sampled node. But if it is the quality problem within the product qualified period, the retailers are able to request the part compensation from the food producers.

### The contagion model of food safety risk

#### Decision model of the food retailers in the face of the contagion model of food safety risk

According to the above, we use $H_{pf}$ to mark the probability of the unqualified products. Thus based on the historical trading data between the food retailers and the food manufacturers, the food retailers would estimate the probability of the unqualified products that were provided by the food manufacturers as follow.

1 $$H_{pf}=(1-q_s){\phi _{p}}$$

Thus if the food retailers can obtain the probability of the unqualified products that were provided by the food manufacturers, the food retailers need to submit the food order quantity *R* to the food producers as follow.

2 $$R={\frac{D}{1-{H_{pf}}{g_r}}}$$

where we can obtain the correlation between the order quantity and the demand quantity. At the same time, the food retailers could estimate the order quantity of the unqualified of products and all kinds of cost. Thus the expected return of the food retailers by selling purchased food as follow.

3 $${\mathbb {E}}_r=D{P_{rc}}-D{c_{rs}}-R{P_{pr}}-R{c_s}{q_s}-R{{H_{pf}}} (1-g_r){\beta _1}{\beta _2}{\psi }-R{{H_{pf}}^2}{g_r}{F_r}(1-{\theta })$$

where $R{c_s}{q_s}$ is the food sampling cost of the food retailers. $R{{H_{pf}}}(1-g_r){\beta _1}{\beta _2}{\psi }$ is the compensation of selling unqualified products for the consumers after this behavior is discovered by the consumers. $R{{H_{pf}}^2}{g_r}{F_r}$ is the fine of selling unqualified products that is discovered by the sampling behavior of the government in the retail link of the food retailers. $R{{H_{pf}}^2}{g_r}{F_r}{\theta }$ is the punishment compensation of the food manufacturers for the unqualified products that is discovered by the sampling rate of the government in the retail link of the food retailers. Namely,

4 $$\begin{aligned} {\mathbb {E}}_r=D[{P_{rc}}-{c_{rs}}-{\frac{{P_{pr}}+{c_s}{q_s}+{(1-q_s) {{\phi _{p}}}}(1-g_r){\beta _1}{\beta _2}{\psi }+{(1-q_s)^2{{\phi _{p}}^2}}{g_r}{F_r}(1-{\theta })}{1-{(1-q_s){{\phi _{p}}}}{g_r}}}] \end{aligned}$$

##### **Proposition 1**

*For the food retailers, the optimal sampling rate of the food retailers as follow:*

5 $$\begin{aligned} {q_s} = 1 -\frac{1}{{g_r}{\phi _p}} + \sqrt{\frac{{{P_{pr}}{\phi _p}{g_r} - {c_s}(1 - {\phi _p}{g_r}) + (1 - {g_r}){\phi _p}{\beta _1}{\beta _2}\psi + {\phi _p}{F_r}(1 - \theta )}}{{{\phi _p}^3{g_r}^2{F_r}(1 - \theta )}}},\; and \;{q_s}\in [0,1] \end{aligned}$$

##### *Proof*

According to the Eq. (4), we can get

6 $$\begin{aligned} \frac{{\partial {{\mathbb {E}_r}}}}{{\partial {q_s}}}&= \frac{{D\left[ {{P_{pr}}{\phi _p}{g_r} - {c_s}(1 - {\phi _p}{g_r}) + (1 - {g_r}){\beta _1}{\beta _2}\psi } \right] - D{F_r}{\phi _p}^3{g_r}^2(1 - \theta ) [({1-q_s})^2-\frac{2-2q_s}{{\phi _p}{g_r}}]}}{{{{\left[ {1 - (1 - {q_s}){\phi _p}{g_r}} \right] }^2}}} \end{aligned}$$

7 $$\begin{aligned} \frac{{{\partial ^2}{{\mathbb {E}}_r}}}{{\partial {q_s}^2}}&= \frac{{ - 2D{\phi _p}^2{g_r}{F_r}(1 - \theta )}}{{{{\left[ {1 - (1 - {q_s}){\phi _p}{g_r}} \right] }^3}}} \end{aligned}$$

According to the Eq. (7), we can get $\frac{{{\partial ^2}{{\mathbb {E}}_r}}}{{\partial {q_s}^2}} \le 0$. Thus the function ${\mathbb {E}_r}$ of the sampling rate $q_s$ of the food retailers is a concave function.

According to the above, if the food retailers want to maximize their profit, the sampling rate $q_s$ of the food retailers must satisfy $\frac{{\partial {\mathbb {E}_r}}}{{\partial {q_s}}} =0$. Thus we can get the Eq. (5). $\square$

#### Decision model of the food retailers in the face of the contagion model of food safety risk

According to the behavior of the food retailers and the historical trading data between the food retailers and the food manufacturers, we can obtain the mean $\phi _{r}$ of the sampling rate of the food retailers in the nearly three phase, and estimate the sampling rate $g_s$ of the government in the production link of the food producers. Thus for rational food manufacturers, they would abandon the behavior of the adulteration and provide qualified food when $R \leqslant Q$. The reason is that the behavior of the adulteration cannot increase income of the food manufacturers, and wold reduce earnings of the food manufacturers. When $R>Q$, the food manufacturers will increase the supply of food by adulterate inferior materials, which further increase earnings of the food manufacturers. Thus when $R \leqslant Q$, the expected return of the food manufacturers as follow.

8 $${\mathbb {E}_p} = R({P_{pr}} - {c_p} - {c_{pp}})$$

When $R>Q$, the relationship between the food purchase orders of the food retailers and the food supply of the food manufacturers is

9 $$R=Q(1 - {q_p})+ Q{q_p}(1 + \alpha )$$

Thus when $R>Q$, the expected return of the food manufacturers as follow

10 $${\mathbb {E}_p} = R{P_{pr}} - R({c_p} + {c_{pp}}) - Q{q_p}^2(1 + \alpha ){g_p}{F_p} - R{H_{pf}}(1 - {g_p})(1 - {\phi _r}){q_p}{g_r}{F_r}\theta$$

Namely,

11 $${\mathbb {E}_p} = Q(1 + {q_p}\alpha )\left[ {({P_{pr}} - {c_p} - {c_{pp}}) - (1 - {q_s})(1 - {g_p})(1 - {\phi _r}){\phi _p}{q_p}{g_r}{F_r}\theta } \right] - Q{q_p}^2(1 + \alpha ){g_p}{F_p}$$

##### **Proposition 2**

*For the food manufacturers, the optimal raw material adulteration rate of the food manufacturers as follow:*

12 $$\begin{aligned} {q_p} = \frac{{\alpha ({P_{pr}} - {c_p} - {c_{pp}}) - (1 - {q_s})(1 - {g_p})(1 - {\phi _r}){\phi _p}{g_r}{F_r}\theta }}{{2\alpha (1 - {q_s})(1 - {g_p})(1 - {\phi _r}){\phi _p}{g_r}{F_r}\theta + 2(1 + \alpha ){g_p}{F_p}}},\quad and\quad {q_P} \in [0,1] \end{aligned}$$

##### *Proof*

According to the Eq. (11), we can get

13 $$\begin{aligned} \frac{{\partial {\mathbb {E}_p}}}{{\partial {q_p}}}&= Q\alpha ({P_{pr}} - {c_p} - {c_{pp}}) - Q(1 + 2{q_p}\alpha )(1 - {q_s})(1 - {g_p})(1 - {\phi _r}){\phi _p}{g_r}{F_r}\theta - 2Q{q_p}(1 + \alpha ){g_p}{F_p} \end{aligned}$$

14 $$\begin{aligned} \frac{{{\partial ^2}{\mathbb {E}_p}}}{{\partial {q_p}^2}}= & {} - 2Q\alpha (1 - {q_s})(1 - {g_p})(1 - {\phi _r}){\phi _p}{g_r}{F_r}\theta - 2Q(1 + \alpha ){g_p}{F_p} \end{aligned}$$

According to the Eq. (14), we can get $\frac{{{\partial ^2}{\mathbb {E}_p}}}{{\partial {q_p}^2}} \le 0$. Thus the function ${\mathbb {E}_p}$ of the raw material adulteration rate $q_p$ of the food manufacturers is a concave function.

According to the above, if the food manufacturers want to maximize their earnings, the raw material adulteration rate $q_p$ of the food manufacturers must satisfy $\frac{{\partial {\mathbb {E}_p}}}{{\partial {q_p}}} =0$. Thus we can get the Eq. (12).

## The simulation analysis of the contagion model of food safety risk under the disturbance of the supply and demand

In the spread model of food safety risk, the risk behaviors of the food manufacturers will be spread along with the food supply chain. In the spread process of food safety risk, raw materials suppliers, food producers, food retailers, government regulators, and consumers will make different behaviors to optimize their goals for meeting their purposes under the different circumstances. However, given the absence of a large amount of time series data for empirical tests, numerical simulation analysis is the most effective testing method. Such analysis is conducted by considering the different values of the parameters in the spread model of food safety risk under the disturbance of the supply and demand. The following are assumed: $F_p=500$, $F_r=800$, $P_{pr}=60$, $C_p=30$, $C_s=40$, $C_{pp}=10$, $\alpha =2$, $\phi _p=0.15$, $\phi _r=0.35$, $\theta =0.3$, $\psi =1200$, $D=10,000$, $Q=10,000$. Thus we use simulation analysis to find the spreading rules of food safety risk and behavioral strategy selection of the food producers and the food retailers under the disturbance of the supply and demand.

### The risk contagion mechanism of unsafe food and the behavioral choice of the food producers and the food retailers

**Fig. 2:**
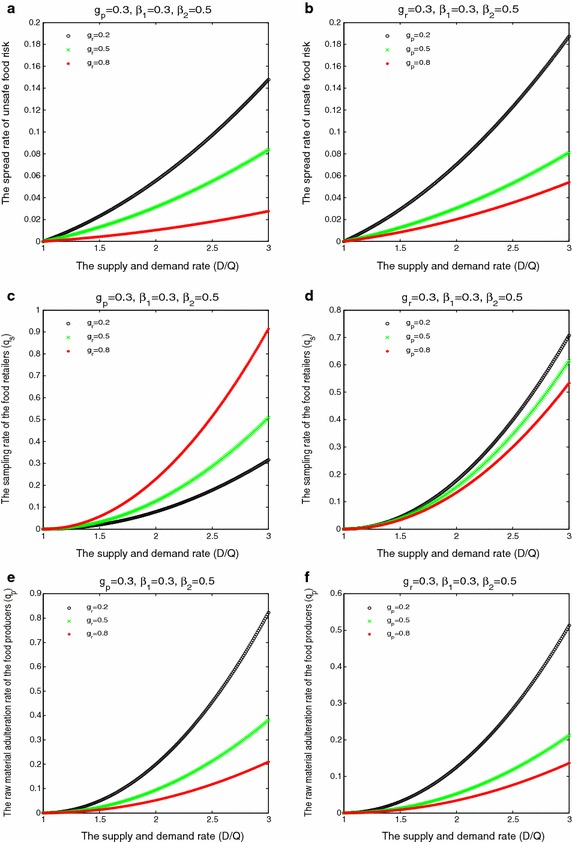
The influencing mechanism of the supply-demand relationship and government supervision on the risk spread of food safety and the behaviors of the food producers and the food retailers. **a**, **b** the effect of the supply-demand relationship on the spread rate of unsafe food risk under different government supervision behaviors; **c**, **d** the effect of the supply-demand relationship on the sampling rate of the food retailers under different government supervision behaviors; **e**, **f** the effect of the supply-demand relationship on the raw material adulteration rate of the food producers under different government supervision behaviors

In Fig. 2, we described the influencing mechanism of the supply-demand relationship and government supervision on the risk spread of food safety and the behaviors of the food producers and the food retailers. From Fig. 2a, b, under the constraints of fixed price and cost, with the increase in the imbalance of the supply-demand relationship, the risk spread rate of unsafe food appeared the phenomenon of accelerated increasing. However, due to the existing of government supervision, the risk spread rate of unsafe food is difficult to reach the upper bound 20 %. And the effects of the behaviors of government supervision are more significant to control the risk spread of unsafe food. In addition, under the lower compensation ratio of the food producers to the food retailers if the unqualified products are found by the government in the retail link, the effect of the sampling behavior of the government in the retail link on controlling the risk spread of unsafe food is more significant than the sampling behavior of the government in the production link. This reason is that the sampling behavior of the government in the retail link strengthened the sampling rate of the food retailers, and which will reduce the raw material adulteration rate of the food producers. In Fig. 2c, d, we found that, with the increase in the degree of the imbalance of the supply-demand relationship, the sampling rate of the food retailers accelerated increasing. And the sampling behavior of the government in the retail link promoted the sampling rate of the food retailers, but the sampling behavior of the government in the production link weakened the sampling rate of the food retailers. This implied that the food retailers had the typical adverse selection behavior for the sampling behavior of the government in the production link. This also verify the above results Fig. 2a, b again. In Fig. 2e, f, with the increase in the degree of the imbalance of the supply-demand relationship, the raw material adulteration rate of the food producers accelerated increasing. However, government supervision behaviors can effectively controlled the raw material adulteration behavior of the food producers, and significantly reduced the raw material adulteration rate of the food producers. And the effect of the sampling behavior of the government in the production link on controlling and weakening the raw material adulteration behavior of the food producers is more significant than the sampling behavior of the government in the retail link.

**Fig. 3:**
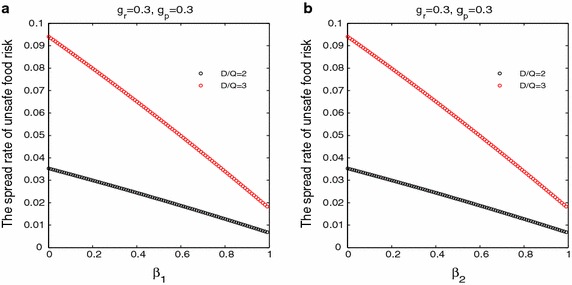
**a** The effect of the probability of taking legal action to defend their rights and interests after consumers find unqualified products on the spread rate of unsafe food risk; **b** the effect of the probability of winning after consumers take legal action on the spread rate of unsafe food risk

In Fig. 3, with the increase in the probability of taking legal action to defend their rights after consumers find unqualified products and the probability of winning after consumers take legal action on the risk spread of unsafe food, the risk spread rate of unsafe food appeared the downward trend. This implied that intensifying the awareness of consumer rights protection and enhancing in the level of legal protection for consumer rights can weakened in certain extent the risk spread of unsafe food. This also implied that intensifying the awareness of consumer rights protection and enhancing in the level of legal protection of consumer rights effectively increased the pressure on the internal control of food safety for the food producers and the food retailers, and promoted them to produce or sell more safe food production. Thus the government should build effective system of consumer rights protection, and inspire consumer taking legal action to defend their rights and interests when they finding unqualified products.

### The effect of the regulation behavioral choice of government on the contagion rate of unsafe food risk and the behaviors of the food producers and the food retailers

**Fig. 4:**
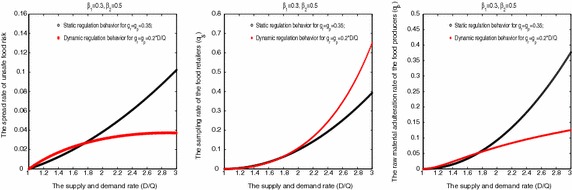
The influencing mechanism of the supply-demand relationship on the risk spread of food safety and the behaviors of the food producers and the food retailers under the static regulation behavior and the dynamic regulation behavior

In Fig. 4, we assumed that the government will have two kinds of regulation behavioral choices: the static regulation behavior and the dynamic regulation behavior. And let the parameter $g_r=g_p=0.35$ of the static regulation behavior and the parameter $g_r=g_p=0.2*D/Q$ of the dynamic regulation behavior. Thus we found that the effect of the dynamic regulation behavior is more significant than the static regulation behavior under the imbalance of the supply-demand relationship. In Fig. 4a, relative to the static regulation behavior where the dynamic regulation behavior more significantly decreased the risk spread rate of unsafe food. In Fig. 4b, relative to the static regulation behavior where the dynamic regulation behavior more significantly increased the sampling rate of the food retailers. In Fig. 4c, relative to the static regulation behavior where the dynamic regulation behavior more significantly decreased the raw material adulteration rate of the food producers.

## Conclusion

In this paper, we design a food safety risk spread model from food producer-to-consumer in the food supply chain based on the the imbalance of the supply-demand relationship of food. We use theoretical analysis and numerical simulation to describe the influence and active mechanism of the supply-demand relationship and government supervision behaviors on the risk spread of food safety and the behaviors of the food producers and the food retailers. We also analyze the effect of the awareness of consumer rights protection and the level of legal protection of consumer rights on the risk spread of food safety. The theoretical analysis and numerical simulation result showed that, (1) with the increase in the imbalance of the supply-demand relationship, the risk spread rate of unsafe food appeared the phenomenon of accelerated increasing. Thus stabilize the market supply-demand relationship of food is most important part of government regulatory. (2) the behaviors of government supervision behaviors and strategy choice more significant effect on controlling the risk spread of unsafe food, enhancing the sampling rate of the food retailers, and decreasing the raw material adulteration rate of the food producers. Thus the government should strengthen the many-links supervision of food supply chain. (3) intensifying the awareness of consumer rights protection and enhancing in the level of legal protection of consumer rights effectively decreased the risk spread rate of unsafe food. Thus the government should build effective system of consumer rights protection, and inspire consumer taking legal action to defend their rights and interests when they finding unqualified products. Certainly, this model and numerical simulation also have certain scope of application and limitations, for example the variable design and parameter value selection, and the demand elasticity characteristics of food productions and so on. Thus we will further deepen and expand in future study.
